# “Cannot ventilate, cannot intubate” situation after penetration of the tongue root through to the epipharynx by a surfboard: a case report

**DOI:** 10.1186/s13256-017-1284-5

**Published:** 2017-05-01

**Authors:** Yuko Ono, Miha Kunii, Tomohiro Miura, Kazuaki Shinohara

**Affiliations:** 1 Emergency and Critical Care Medical Center, Fukushima Medical University Hospital, 1 Hikarigaoka, Fukushima, Fukushima 960-1295 Japan; 2 Department of Anesthesiology, Ohta General Hospital Foundation, Ohta Nishinouchi Hospital, 2-5-20 Nishinouchi, Fukushima, Fukushima 963-8558 Japan; 3 Department of Otolaryngology, Fukushima Medical University, 1 Hikarigaoka, Fukushima, Fukushima 960-1295 Japan; 4 Department of Head and Neck Surgery, Ohta General Hospital Foundation, Ohta Nishinouchi Hospital, 2-5-20 Nishinouchi, Fukushima, Fukushima 963-8558 Japan

**Keywords:** Emergency surgical airway, Hypovolemic shock, Penetrating neck injury, Upper airway trauma

## Abstract

**Background:**

Surfing is an increasingly popular activity and surfing-related injuries have increased accordingly. However, to the best of our knowledge, there are no reports of penetrating upper airway injuries in surfers. We present a “cannot ventilate, cannot intubate” situation following penetrating neck injury by a surfboard fin.

**Case presentation:**

A previously healthy 29-year-old Japanese man was swept off his board by a large wave and his left mandible, tongue root, and right epipharynx were penetrated by the surfboard fin. He presented with severe hypovolemic shock because of copious bleeding from his mouth. Direct laryngoscopy failed, as did manual ventilation, because of the exacerbated upper airway bleeding and distorted upper airway anatomy. Open cricothyrotomy was immediately performed, followed by surgical exploration, which revealed extensive ablation of his tongue root and laceration of his lingual artery. After definitive hemostasis and intensive care, he returned home with no sequelae.

**Conclusions:**

The long, semi-sharp surfboard fin created both extensive crushing upper airway lesions and a sharp vascular injury, resulting in a difficult airway. This case illustrates that surfing injuries can prompt a life-threatening airway emergency and serves as a caution for both surfers and health care professionals.

## Background

Surfing is an increasingly popular outdoor activity, with more than 10 million surfers worldwide [1]; however, this sport is not without risks. The estimated incidence of surfing-related injuries is 3.5 to 6.6 per 1000 days [2, 3]. Consequently, health care professionals are likely to encounter patients with surfing-associated trauma. Previous reports described the features of surfing-related craniofacial trauma [4–6], ocular injury [7], spinal injury [4–6, 8], and musculoskeletal injuries, including sprains, dislocations, and fractures [4–6]. However, to the best of our knowledge, there are no reports describing penetrating upper airway injuries caused by a surfboard.

We present a case of a surfer who had a penetrating injury of his left mandible, tongue root, and right epipharynx caused by the fin of his surfboard. Both manual ventilation and endotracheal intubation (ETI) were extremely difficult because of the upper airway distortion and massive bleeding. We describe the characteristics and airway management experience of surfboard-associated upper airway trauma.

## Case presentation

**Fig. 1:**
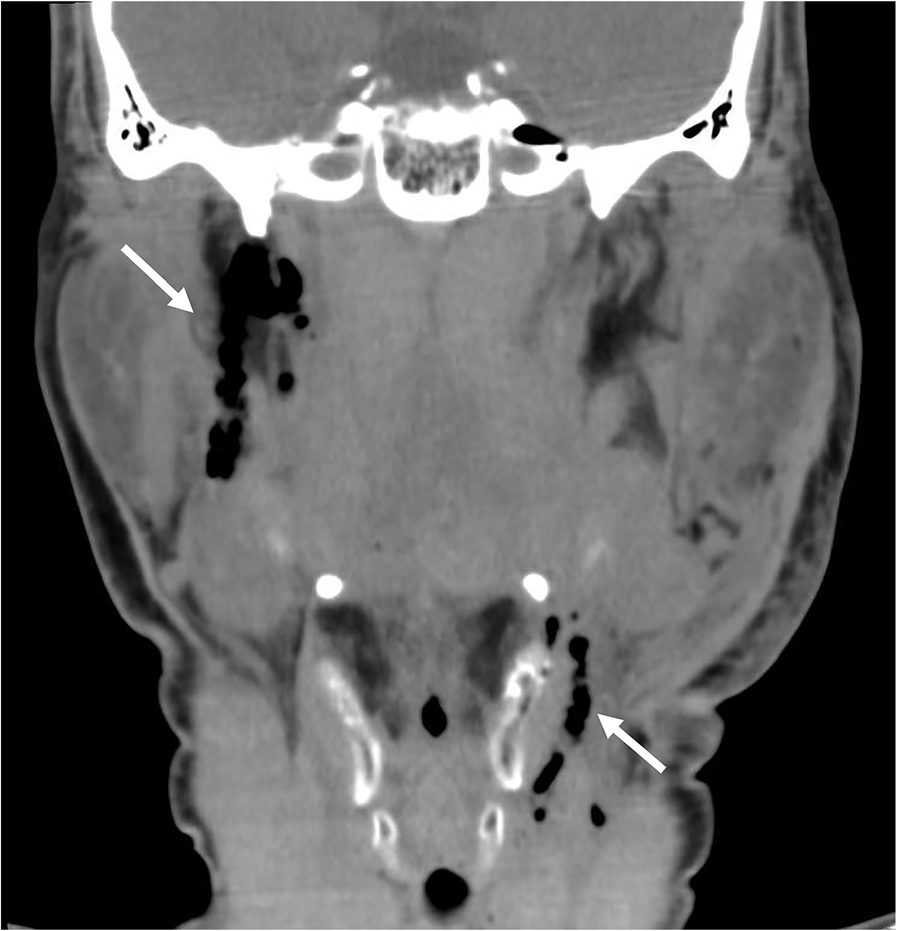
Computed tomography scan showing air in the regions of the left mandible and right styloid process of the temporal bone (*white arrows*), suggesting a penetrating neck injury extending to the skull base

A previously healthy 29-year-old Japanese man who was an experienced surfer was swept off his surfboard by a large wave. He fell on the semi-sharp fin of his own board, which penetrated his left mandibular region through to his right epipharynx. On admission to our emergency department (ED), he was pale and in obvious distress. On physical examination, there was active bleeding from his mouth and a laceration overlying his left mandible. Manual pressure hemostasis was unsuccessful and he developed serious hypovolemic shock. His initial vital signs recorded in the ED were as follows: body temperature, 34.5 °C; heart rate, 126 beats/minute; blood pressure, 72/40 mmHg; respiratory rate, 30 breaths/minute; and percutaneous oxygen saturation (SpO_2_), 93% (on 10 L/minute oxygen via a non-rebreathing mask). He was restless, with a consciousness level of 9 on the Glasgow Coma Scale (E2V2M5). His extremities were cool and damp, but no trauma was evident. He had a well-developed stature but neither a short neck nor micrognathia, and showed no signs of a restricted mouth opening. His cricothyroid membrane was palpable and there were no neck deformities. His physical examination, including assessment of thorax, abdomen, and pelvis, was otherwise normal. He had no history of medication use or allergies. Computed tomography revealed a penetrating soft tissue injury extending from his left mandible to the right styloid process of his temporal bone (Fig. 1; image obtained after securing the airway and temporal hemostasis). Blood gas analysis (on 10 L of oxygen/minute via a non-rebreathing mask) showed hypoxemia and respiratory as well as metabolic acidosis: pH, 7.31; partial pressure of carbon dioxide (pCO_2_), 48.0 mmHg (6.4 kPa); partial pressure of oxygen (pO_2_), 66.6 mmHg (8.9 kPa); bicarbonate (HCO_3_^−^), 22.8 mmol/L; and base excess, −3.0 mmol/L. laboratory data revealed anemia (hemoglobin, 9.5 g/dl; hematocrit, 28.5%) and prolonged prothrombin time (percentage of standard value, 60.2%).

Combining these findings, the most likely diagnosis was hypovolemic shock arising from the penetrating left mandibular region injury, which reached our patient’s right skull base and corresponded to a zone III penetrating neck injury [9]. Hypoxemia, confirmed on blood gas analysis, may have exacerbated the tissue hypoperfusion.

The need for immediate definitive airway management and surgical hemostasis was apparent. As a difficult airway was strongly predicted in this case, a surgical airway device was prepared before ETI was attempted. Tracheotomy under local anesthesia was not considered because he was restless and was unlikely to have tolerated this procedure. After the intravenous administration of fentanyl (1 μg/kg) and ketamine (1 mg/kg), direct laryngoscopy was performed; however, no portion of his epiglottis could be confirmed (Cormack-Lehane grade 4 view) because of the upper airway distortion and copious bleeding. This attempt resulted in both a further deterioration of his upper airway and increased hemorrhage, such that continued manual ventilation was unsuccessful. The rapid four-step cricothyrotomy technique [10] was performed immediately, resulting in establishing a definitive airway using a cuffed endotracheal tube (inner diameter, 6.5 mm). This technique was completed within 40 seconds and allowed the placement of oropharyngeal gauze packing and acute hemostasis. Oxygen (10 L/minute) was administrated using a two-handed technique with a Jackson Rees circuit before and during cricothyrotomy; however, SpO_2_ decreased to <80% before we could establish a definitive airway. Fortunately, other airway-related adverse events including cardiac arrest, dysrhythmia, and hypotension did not occur.

**Fig. 2:**
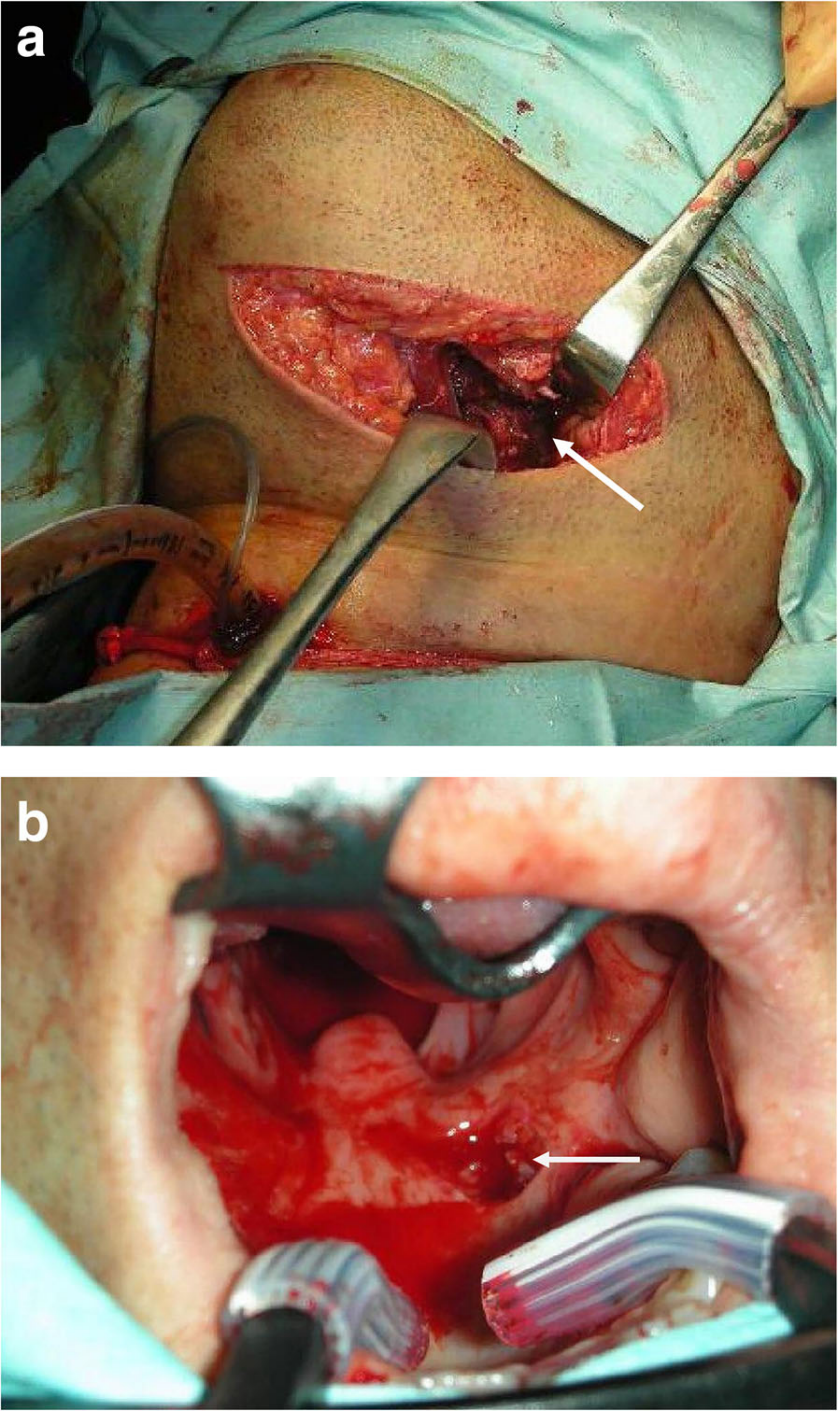
Operative findings of a penetrating neck injury caused by a surfboard fin. **a** Wounds caused by entry of the fin from the left mandibular region were explored surgically. *White arrow* indicates the ruptured left digastric and tongue muscles. **b** Oral view. The surfboard fin penetrated the right soft palate (*white arrow*) and reached the right parapharyngeal space

Surgical exploration revealed laceration of his left digastric muscle, submandibular gland, and lingual muscles (Fig. 2a). The left root of his tongue and right palatine tonsil were crushed extensively. The wound extended from the left submandibular space to the right parapharyngeal space (Fig. 2b), but there was no evidence of skull base fracture or leakage of cerebrospinal fluid. The major source of bleeding was from a sharp laceration to his lingual artery, with additional oozing from the ablated root of his tongue and palatine tonsil. The former was ligated and, later, electrically coagulated. After copious irrigation and wound repair, the cricothyrotomy was converted to a tracheotomy. The volume of intraoperative blood loss was 1005 ml, and he received 18 units of packed red blood cells, 10 units of fresh frozen plasma, and 20 units of platelet concentrate. Once these procedures were complete, he was admitted to our intensive care unit where controlled ventilation was continued.

Mechanical ventilation was discontinued on hospital day 3. The tracheotomy tube was successfully removed on day 10, and the tracheotomy site was then closed. The following day our patient reported dysphagia, which was attributed to hypoglossal nerve injury. After receiving active rehabilitation, he was able to eat and drink without choking or coughing, and he was discharged home without nursing care on hospital day 22. At his out-patient follow-up visit 2 months later, the dysphagia had resolved completely, and he had no emergency surgical airway-related complications, including hoarseness or subglottic stenosis.

## Discussion

We describe the characteristics of a surfboard-associated penetrating upper airway trauma and the subsequent airway management in this patient. Head and neck injuries secondary to surfing are well recognized [4–6]. However, the injuries are typically superficial lacerations, skull and facial fractures, or cervical spine injuries [4–6]. This case adds to the literature on the life-threatening airway emergencies that can develop following a surfing injury.

The long, semi-sharp surfboard fin caused both an extensive crushing upper airway lesion and a sharp vascular laceration. The former, involving the left tongue root and right palatine tonsil, was similar to impalement injuries by other semi-sharp objects, such as a tree branch [11], pieces of metal [12], and wire [13]. Repeated ETI attempts may cause complete airway obstruction, with a devastating outcome. In fact, in our patient, the one direct laryngoscopy attempt exacerbated the upper airway hemorrhage and made further manual mask ventilation impossible. Our patient also had massive upper airway bleeding from a sharp laceration of his lingual artery, which prevented the use of a supraglottic airway device, video laryngoscope, or flexible fiberoptic bronchoscopy. The only option in this situation was an emergency surgical airway. Cogbill *et al*. [14] reported the need for emergency cricothyrotomy in 8% and tracheotomy in 6% of patients with severe upper airway hemorrhage. As demonstrated in the current case and in their study, health care professionals should prepare for an emergency surgical airway before attempting ETI in patients with uncontrolled oropharyngeal hemorrhage.

We used the rapid four-step technique [10] to establish a definitive airway in our patient. The bougie-assisted cricothyrotomy technique described by Hill *et al*. may be faster and easier than the rapid four-step technique, especially in inexperienced hands [15]. Percutaneous cricothyrotomy is a reported alternative to open cricothyrotomy [16]; however, the percutaneous technique has severe potential complications, such as pretracheal false passage, and hypopharyngeal and esophageal intubation [17]. Therefore, we preferentially perform open cricothyrotomy as our rescue strategy.

Our patient experienced penetration of the left tongue root through to the right skull base, consistent with a zone III penetrating injury of the neck [9]. In these cases, hemostasis is extremely difficult because of the anatomical complexity of the injury site [9]. An advantage of an emergency surgical airway is that it allows separation of the artificial airway and surgical field. In our patient, cricothyrotomy allowed oropharyngeal gauze packing and acute hemostasis, thereby providing a clear surgical field. This advantage is especially important in patients with severe hypovolemic shock.

In our patient, oxygen (10 L/minute) was administered using a two-handed technique with a Jackson Rees circuit before and during the cricothyrotomy, but it was not sufficient to prevent hypoxemia. Recently, Patel and Nouraei reported that transnasal humidified high-flow oxygenation insufflation successfully extended the apnea time of 25 patients with difficult airways [18]. As transnasal high-flow oxygenation has also been reported to be an effective and feasible option to treat acute respiratory failure in the ED [19], this technique may be a promising alternative for oxygenation in patients similar to ours.

## Conclusions

In this report, we discussed our experience treating a patient with surfing-related penetrating upper airway injuries through the left mandibular region to the right parapharyngeal space. The long, semi-sharp surfboard fin created both extensive crushing upper airway lesions and a sharp vascular laceration, resulting in a difficult airway. This case illustrates that surfing injuries can prompt a life-threatening airway emergency and serves as a caution for both surfers and health care professionals.

## Abbreviations

ED: Emergency department
ETI: Endotracheal intubation
SpO_2_: Percutaneous oxygen saturation
